# One in Five Patients Returns to Sport Before Assessment: Real-World Use of the Ankle-GO Composite Score in 6934 Patients

**DOI:** 10.3390/jcm15145463

**Published:** 2026-07-13

**Authors:** Ronny Lopes, Alexandre Hardy, François Fourchet

**Affiliations:** 1 Centre Orthopédique Santy, FIFA Medical Center of Excellence, Hôpital Privé Jean Mermoz, Groupe GDS-Ramsay, 24 Avenue Paul Santy, 69008 Lyon, France; 2 Clinique du Sport Paris, 75005 Paris, France; 3 Physiotherapy Department, La Tour Hospital, Swiss Olympic Medical Center, 1217 Meyrin, Switzerland; francois.fourchet@metamorffose.com; 4 Groupe Maisonneuve SA, Avenue de Châtelaine 60–64, 1219 Geneva, Switzerland

**Keywords:** ankle sprain, chronic lateral ankle instability, return to sport, composite score, real-world data, digital health, Ankle-GO

## Abstract

**Background**: The Ankle-GO is a composite score integrating physical, functional, and psychological recovery after lateral ankle sprain (LAS). Its psychometric properties have been validated prospectively, but no large-scale data describe how it is used in routine practice. We aimed to characterise real-world usage patterns, score distributions, and the gap between intended and actual clinical use. **Methods**: Retrospective observational analysis of all Ankle-GO web-application sessions recorded between 1 February and 31 December 2024. Patients with at least one valid session and a single injured ankle were included. Quantitative variables were summarised as mean (SD) or median (IQR); proportions are reported with 95% Wilson confidence intervals (CIs). No inferential testing was performed. **Results**: Of 8988 registered patients, 6934 (77.1%) were analysed; 88.5% were assessed after ankle sprain (mean age 26.0 ± 10.0 years; 92.5% in France). Only 14.0% of sessions were follow-ups. Injured-ankle scores were consistently lower than contralateral scores and improved modestly across sessions. Notably, 20.4% (95% CI 19.4–21.4) of patients had already returned to sport before their first assessment. **Conclusions**: Real-world Ankle-GO implementation diverges substantially from its intended prospective use, defining clear priorities for future standardised, outcome-driven studies.

## 1. Introduction

Lateral ankle sprain (LAS) is the most common musculoskeletal injury in sports and a leading cause of emergency department visits in both the United States and Europe. In the United States, the incidence of ankle sprains is 2.2 per 1000 person-years, peaking between 15 and 19 years of age [1]. European studies report comparable incidence rates of 5.3 to 7.0 per 1000 person-years, with an estimated 6000 ankle sprains per day in France alone [2,3]. Ankle sprains and fractures also generate substantial direct healthcare costs and indirect costs related to productivity loss [4]. Separately, published international data indicate that up to 33% of patients may experience residual pain at one year, recurrence may affect up to 34% within three years, and approximately 40% may progress to chronic ankle instability (CAI) [5,6]. The wide reported range of full recovery (36–85%) reflects heterogeneity in injury severity, treatment pathways, and definitions of recovery. CAI is associated with persistent functional deficits, repeated giving-way episodes, and substantial costs, and may ultimately require surgical ligament reconstruction [7,8].

Premature return to sport (RTS) is a recognised risk factor for recurrent sprains, yet 86% of high-school athletes return to sport within one week of an acute LAS despite ligament healing times of six to twelve weeks [9]. However, the pathway leading to RTS differs according to the sporting context: in professional athletes, RTS is usually a multidisciplinary decision involving medical, rehabilitation, coaching, and performance staff, whereas in recreational or non-professional athletes it may be more patient-driven and less formally supervised. Regardless of the setting, RTS decisions should ideally be criteria-based and include restoration of pain-free function, ankle range of motion, strength, balance, sport-specific performance, and psychological readiness [10,11]. Standardised composite assessments may therefore help identify residual deficits or incomplete rehabilitation before RTS.

The Ankle-GO score was developed to address the lack of a standardised, criteria-based tool to support return-to-sport decision-making after lateral ankle sprain and chronic ankle instability [10,11,12,13]. It combines patient-reported function and psychological readiness with balance and hop-performance tests, including the FAAM, ALR-RSI, SLS, SEBT, SHT, and F8T [12,14,15,16,17,18]. In principle, the Ankle-GO is intended to be administered by clinicians, most commonly physiotherapists or sports medicine practitioners, during the rehabilitation pathway when return to sport is being considered, rather than after the athlete has already returned. The timing of testing is therefore a clinical decision based on injury severity, symptom evolution, rehabilitation progress, and sport-specific demands. Subsequent studies have applied the Ankle-GO to other ankle pathologies, including ankle ligament reconstruction and Achilles tendon repair, at pathology-specific follow-up time points [19,20]. An Ankle-GO score below 8 points at the time of return to sport has also been associated with an increased risk of recurrent lateral ankle sprain within two years [21].

Recent reviews in the Journal of Clinical Medicine have positioned the Ankle-GO among the most promising emerging tools for monitoring outcomes after ankle ligament reconstruction [22] and have repeatedly emphasised the persistent lack of consensus on RTS criteria after LAS [23,24].

Despite this growing evidence base derived from controlled cohorts, almost nothing is known about how the Ankle-GO is actually used in routine clinical practice. Since the Ankle-GO web application was launched in February 2024, several thousand patients have been assessed by physiotherapists across multiple countries, generating a unique opportunity to characterise real-world implementation. Recent work has shown that adherence of physiotherapists to ankle-sprain clinical practice guidelines remains highly variable, suggesting that the gap between recommended and actual practice deserves explicit study [25].

In clinical practice, the Ankle-GO is mainly used in French-speaking European settings, particularly in France, Switzerland, and Belgium. Its purpose is to support criteria-based return-to-sport decision-making by identifying residual physical, functional, and psychological deficits after lateral ankle sprain. However, the score does not provide information on the treatment pathway received before testing. Therefore, a low or incomplete Ankle-GO profile may reflect persistent impairment, but also incomplete, heterogeneous, or non-standardized prior rehabilitation.

The present study therefore had two specific aims: (i) to provide a large-scale descriptive overview of patient demographics, session characteristics, score distributions, and subtest performance from the Ankle-GO database; and (ii) to quantify the extent to which the tool is used prospectively (before RTS) versus retrospectively (after RTS), as the latter represents a deviation from its intended purpose. This study does not aim to validate the Ankle-GO for RTS decision-making, but rather to inform the design of future prospective, outcome-driven studies and the development of standardised implementation guidelines.

## 2. Materials and Methods

### 2.1. Study Design and Ethics

This was a retrospective, observational, descriptive analysis of routinely collected data from the Ankle-GO web application. The study was conducted in accordance with the Declaration of Helsinki and reported following the Strengthening the Reporting of Observational Studies in Epidemiology (STROBE) statement. The protocol was reviewed by the institutional ethics committee of Ramsay Santé, which granted a formal waiver of full ethical approval given the retrospective and pseudonymised nature of the dataset (reference COS-RGDS-2026-04-011-LOPES). All patients provided electronic informed consent to the secondary use of their data upon registering in the web application. Data were hosted by KOOKLINE (Sainte-Luce-sur-Loire, France) on Niwanet servers, certified ISO/IEC 27001 for the hosting of personal health data (certificate CT-ISMS-072024-0CU01190), in compliance with the European General Data Protection Regulation (GDPR) and the French national reference methodology MR-004.

### 2.2. Data Source and Study Period

All sessions recorded in the Ankle-GO web application between 1 February 2024 and 31 December 2024 were eligible. The database extraction was performed on 1 January 2025. The Ankle-GO web application is freely available in French and English, with the entire six-subtest battery typically completed in approximately 20 min.

### 2.3. Inclusion and Exclusion Criteria

All patients of any age and country of residence registered during the study period were eligible. Patients were included in the analysis if they had completed at least one Ankle-GO session and had a single injured ankle. We intentionally retained a heterogeneous spectrum of ankle pathologies (sprains, fractures, postoperative conditions, Achilles tendon repairs, and asymptomatic athletes assessed for screening) because the explicit objective was to describe real-world use, not to draw pathology-specific conclusions. The implications of this pooling are discussed extensively in the Section 4.5.

Sessions were excluded if any of the following pre-specified criteria were met: (i) at least one implausible measure, defined as any individual subtest result outside its physiological range (e.g., FAAM ADL > 84 points; FAAM Sport > 32 points; ALR-RSI > 120 points; SLS errors > 30; SEBT reach > 200% of leg length; SHT or F8T completion time < 5 s or > 30 s, as these thresholds correspond to the published 0.5th and 99.5th percentiles of healthy reference data [12,14,15,16,17,18]); (ii) a test evaluation, i.e., a fitness assessment of an asymptomatic athlete or a test run that did not correspond to a genuine single-injured-ankle assessment; (iii) implausible demographic data (age < 5 or >90 years; height < 100 or >230 cm; weight < 20 or >200 kg); (iv) inconsistent injury date(s) recorded for the same patient; (v) bilateral injury or bilateral healthy status recorded for the same patient.

**Figure 1:**
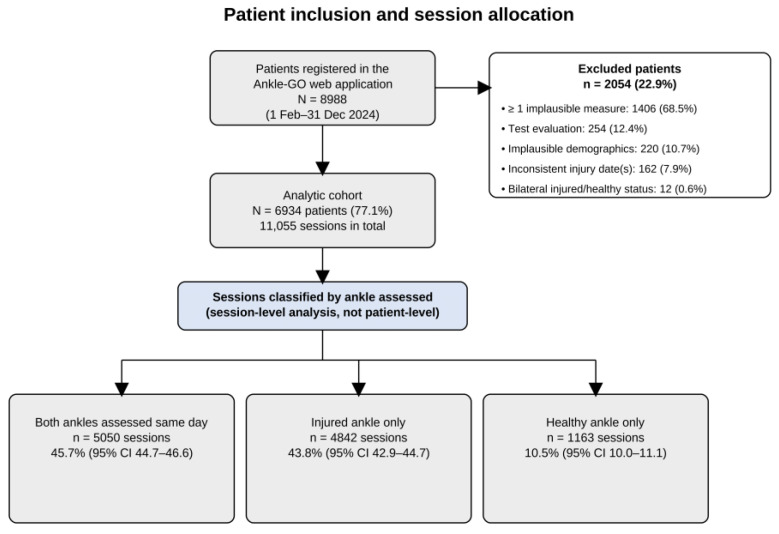
***Flowchart of patient inclusion.*** *Of 8988 patients registered in the Ankle-GO web application between 1 February and 31 December 2024, 2054 (22.9%) were excluded because of at least one implausible measure (n = 1406), test evaluation (n = 254), implausible demographic data (n = 220), inconsistent injury date(s) (n = 162), or bilateral injured/healthy status (n = 12). The final analytic cohort included 6934 patients (77.1%), who generated 11,055 sessions. Sessions were then classified according to whether both ankles, the injured ankle only, or the healthy ankle only were assessed.*

Exclusion subtotals are reconciled in the flowchart (Figure 1) and were as follows: at least one implausible measure (*n* = 1406, 68.5% of all exclusions); test evaluation (*n* = 254, 12.4%); implausible demographic data (*n* = 220, 10.7%); inconsistent injury date(s) (*n* = 162, 7.9%); and bilateral injured or bilateral healthy ankles (*n* = 12, 0.6%). Test evaluations comprised fitness assessments of asymptomatic athletes and test runs that did not meet the inclusion criterion of a single injured ankle. The five categories sum to the 2054 excluded patients (22.9% of those registered).

### 2.4. Ankle-GO Score and Subtests

All assessments were performed by physiotherapists in their own clinical setting following the standardised written protocol provided with the web application, but no centralised training or quality-assurance procedure was implemented. This is a recognised limitation of the dataset (see Section 4.5). The six subtests were:

FAAM—A 29-item self-reported questionnaire validated in French [14], comprising the activities of daily living subscale (ADL, 21 items, range 0–84) and the sports subscale (8 items, range 0–32). Items are rated on a 5-point Likert scale (4 = no difficulty to 0 = unable). Higher scores indicate better function.

ALR-RSI—A 12-item self-reported scale assessing psychological readiness to return to sport (range 0–120), validated in French for the LAS and ligament-reconstruction populations [15,26].

SLS—Static unipodal balance test with eyes closed and hands on hips, scored by counting errors (eyes opening, stepping, loss of contact) over 20 s [16].

SEBT—Dynamic balance test performed in three directions (anterior, posteromedial, posterolateral); reach distance normalised to leg length, expressed as the mean of the three directions [17,18].

SHT—20 side-to-side hops across two lines 30 cm apart, timed in seconds [11].

F8T—Two consecutive figure-of-8 hopping circuits between two cones placed 5 m apart, timed in seconds [11].

Patients were also asked, for each physical task, whether they experienced apprehension (yes/no). The Ankle-GO composite score was calculated from raw values according to the published weighted algorithm (range 0–25, higher = better) [12]. The web application additionally recorded whether patients had returned to sport and whether they had regained their previous level (self-reported, single-item question with four response categories: no return/return at a lower level/return at the same level/return at a higher level).

No formal stratification was performed according to sprain grade, time since sprain, or time after treatment. Sprain severity grading was not available as a mandatory structured field in the web application, and treatment pathways were not standardised across clinicians. Time since injury was recorded and described at baseline, but was not used to define analytical subgroups, because the study was designed as a descriptive real-world implementation analysis rather than as a pathology-, severity-, or time-specific outcome study.

For interpretation, the Ankle-GO was not considered a binary positive/negative test in the present analysis. Rather, each measure was interpreted according to its expected direction of recovery: higher Ankle-GO composite scores, FAAM scores, ALR-RSI scores, and SEBT reach values indicate more favourable function or readiness; fewer SLS errors and shorter SHT and F8T completion times indicate better performance; absence of apprehension during physical tasks is favourable; and return to sport at the same or a higher level is considered more favourable than no return or return at a lower level. Conversely, lower self-reported or balance scores, more SLS errors, longer hop-test completion times, reported apprehension, and no return or return at a lower level indicate a less favourable clinical profile. These interpretations follow the published Ankle-GO scoring framework and the previously validated component measures [12].

### 2.5. Statistical Analysis

Analyses were performed with SAS v9.4 (SAS Institute, Cary, NC, USA). Quantitative variables are summarised with means and standard deviations (SD) or medians and interquartile ranges (IQR) according to their distribution (visually inspected via histograms and Shapiro–Wilk tests). Proportions are reported with 95% Wilson binomial CIs computed with PROC FREQ. Because the dataset was not designed to support inferential testing of recovery trajectories, we did not perform formal hypothesis testing for between-session or between-ankle comparisons; differences are described in numerical terms only, and the term “significant” is avoided in the Section 3 in line with current reporting recommendations [27]. No multivariable modelling was undertaken, as the dataset was not designed to support inferential analysis.

Missing data were not imputed. The percentage of missing values is reported in Table 1 for every variable, and denominators are explicitly stated wherever a proportion is presented. Sex, in particular, was not a mandatory field in the web application until late 2024 and is missing for 56.2% of the analytic cohort; we therefore report sex-stratified percentages over the *n* = 3038 patients with complete data and explicitly do not extrapolate to the full cohort. The Spearman correlation coefficient between successive injured-ankle scores was used as an indicator of short-term score stability. We acknowledge that a correlation coefficient alone does not allow clinicians to interpret intra-individual change; ideally, a Bland–Altman analysis and a minimum detectable change (MDC) from a dedicated test–retest reliability study would be needed, and this is identified as a priority for future work (Section 4.5).

Ankle-GO scores and subtest results were analysed separately according to whether the assessment was for the injured ankle alone, the healthy ankle alone, or both ankles on the same day. RTS data were analysed only for patients whose injured ankle had been assessed. No analyses were performed to assess RTS safety or reinjury risk, as the dataset was not designed for prospective outcome evaluation.

## 3. Results

### 3.1. Study Population

Of 8988 patients registered in the database between 1 February 2024 and 31 December 2024, 6934 (77.1%, 95% CI 76.2–78.0) met inclusion criteria and constituted the analytic cohort. The full reconciliation of exclusions is shown in Figure 1.

Patient characteristics are presented in Table 1. The cohort was predominantly young (mean age 26.0 ± 10.0 years, range 10–68; 23.1% under 18 years) and of normal body mass index (mean 22.8 ± 3.2 kg/m^2^). Among the 3038 patients with sex recorded, 62.6% (95% CI 60.9–64.3) were male and 37.4% (95% CI 35.7–39.1) female; the high proportion of missing values for sex is discussed in Section 4. The cohort was geographically homogeneous, with 92.5% of patients treated in France and a further 4.9% in Belgium and 2.2% in Switzerland. Lateral ankle sprain was the assessment indication in 88.5% (95% CI 87.6–89.3) of cases; fractures (4.1%), ankle stabilisation surgery (3.7%), Achilles tendon repair (1.0%), and other indications accounted for the remainder. Approximately 23% of patients reported a previous ankle sprain. Most patients (82.4%) participated in sports, of whom 6.1% were classified as professional, 39.3% as competitive, and 37.1% as recreational. The median delay between the index injury and the first Ankle-GO assessment was 2.33 months, with substantial variability across patients (IQR 1.31–4.07 months).

### 3.2. Session Characteristics

**Figure 2:**
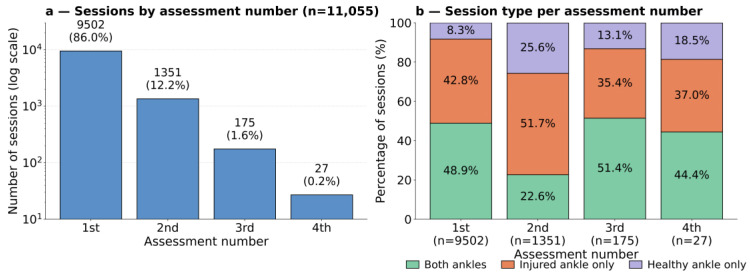
**Session structure.** *(**a**) Distribution of sessions by assessment number (first 86.0%; second 12.2%; third 1.6%; fourth 0.2%). (**b**) Proportion of bilateral versus unilateral assessments by session, showing a fall in bilateral testing from 49% at session 1 to 23% at session 2, stable thereafter.*

The 6934 patients generated 11,055 sessions in total. The vast majority of sessions were first assessments (*n* = 9502; 86.0%, 95% CI 85.3–86.6); only 14.0% (95% CI 13.3–14.6) of sessions were follow-up visits, distributed as second (*n* = 1351; 12.2%), third (*n* = 175; 1.6%), and fourth assessments (*n* = 27; 0.2%) (Figure 2a). The first Ankle-GO assessment was performed at a median of 2.33 months after the index injury (IQR 1.31–4.07), indicating that initial real-world use typically occurred approximately two months post-injury. Subsequent assessments were not scheduled according to a standardized protocol; their timing reflected routine clinical practice and was therefore variable. Approximately half of sessions (45.7%, *n* = 5050) assessed both ankles simultaneously; 43.7% (*n* = 4842) assessed the injured ankle alone, and 10.5% (*n* = 1163) the healthy ankle alone. The proportion of bilateral sessions decreased sharply from 49% at session 1 to 23% at session 2 and remained stable thereafter (Figure 2b). This single-session, predominantly first-assessment pattern is itself a major finding and is interpreted in Section 4.2.

### 3.3. Ankle-GO Composite Scores

**Figure 3:**
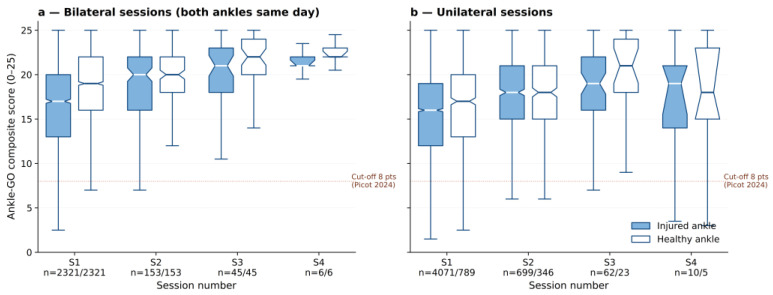
**Ankle-GO composite score by session and laterality.** *Notched box plots (median, IQR) for (**a**) bilateral and (**b**) unilateral injured-ankle sessions. Injured-ankle scores were consistently lower than contralateral healthy-ankle scores at every session, with modest improvement across sessions. The published return-to-sport cut-off* [12] *is indicated. Summary means are reported in* Table 2.

Mean composite scores on the injured ankle were consistently lower than on the contralateral healthy ankle at every session number (Figure 3). For bilateral sessions, mean injured-ankle scores increased from 16.2 ± 4.9 (session 1) to 18.9 ± 4.2 (session 2), 20.2 ± 4.1 (session 3), and 20.5 ± 2.3 (session 4); the corresponding healthy-ankle scores were 18.7 ± 4.0, 20.1 ± 3.3, 21.2 ± 3.3, and 21.8 ± 2.6. For unilateral injured-ankle sessions, mean scores were 15.4 ± 5.1, 17.6 ± 4.8, 18.7 ± 3.4, and 17.6 ± 6.3, respectively. The between-session score gain (≈3–4 points from first to last available session) was smaller than the 5- to 6-point gains reported in published prospective cohorts after ankle ligament reconstruction (4–6 months), Achilles repair (6–9 months), and acute LAS (2–4 months) [12,19,20]. As detailed in Section 4, this difference must be interpreted in the light of (i) heterogeneous timing of the first assessment, (ii) the descriptive (between-cohort) nature of these comparisons, and (iii) the very low rate of repeat assessments.

**Table 2:** Ankle-GO composite scores, score stability, and return-to-sport metrics by session number.

Ankle-GO Composite Score, Mean ± SD	Session 1	Session 2	Session 3	Session 4
Bilateral sessions—injured ankle	16.2 ± 4.9	18.9 ± 4.2	20.2 ± 4.1	20.5 ± 2.3
Bilateral sessions—healthy ankle	18.7 ± 4.0	20.1 ± 3.3	21.2 ± 3.3	21.8 ± 2.6
Unilateral injured-ankle sessions	15.4 ± 5.1	17.6 ± 4.8	18.7 ± 3.4	17.6 ± 6.3
***Return to sport (RTS) and score stability***				
RTS before first assessment, % (95% CI)	20.4 (19.4–21.4)			
Any RTS reported, %	20.4	—	—	43.8 †
Regained previous level among returners, % (95% CI)	45.0 (42.3–47.7)	54.2	57.1	57.1
Score stability, Spearman ρ (sessions 1 ↔ 2, *n* = 526)	0.46 (95% CI 0.39–0.53)			

† Session-4 estimate is based on only 16 patients (95% CI 23.1–66.8) and is statistically fragile. No inferential between-session or between-ankle testing was performed; differences are descriptive only. Healthy-ankle scores were lower than the value of 19.6 reported in the original validation cohort.

The Spearman correlation between Ankle-GO scores at sessions 1 and 2 in patients with both available (*n* = 526) was moderate (ρ = 0.46, 95% CI 0.39–0.53), indicating that early scores partially predict later scores but with substantial inter-individual variability.

### 3.4. Subtest Results

**Figure 4:**
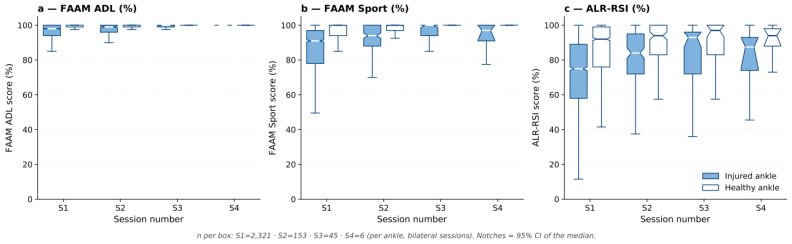
**Patient-reported subtest distributions.** *Notched box plots (median, IQR) for FAAM-ADL, FAAM-Sport, and ALR-RSI on injured versus healthy ankles across sessions. Notched plots are used because FAAM and SLS distributions were strongly left-skewed. ALR-RSI showed the smallest between-session improvement.*

Figure 4 displays subtest distributions as medians and IQRs, with notched box plots, because the FAAM and SLS distributions were strongly left-skewed and could otherwise generate confidence intervals exceeding 100%. For the FAAM ADL subscale, median scores were generally above 80% across all sessions, with smaller variability than for the FAAM Sport subscale, which captured greater between-patient heterogeneity in sports participation. Both subscales showed lower scores on the injured ankle than on the healthy ankle and modest improvement across sessions. ALR-RSI scores were also lower on the injured side; improvements between sessions were smaller than for the FAAM, suggesting that psychological readiness recovers more slowly than self-reported function—a pattern that may help explain why some patients return to sport before reaching high psychological-readiness scores (Section 4.3).

**Figure 5:**
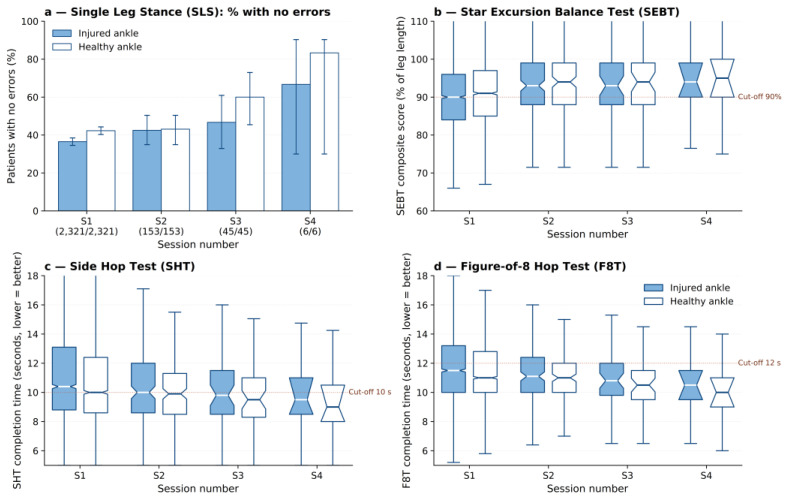
**Functional subtest results.** *Single-Leg Stance (errors), Star Excursion Balance Test (% of leg length), Side Hop Test, and Figure-of-8 Test (seconds) for injured and healthy ankles, by session. Differences between sides and across sessions were small and are described numerically without inferential testing.*

Figure 5 shows results for the four physical tests. At the first session, approximately 40% of patients completed the SLS with no errors on the injured ankle, with progressive improvement across sessions and consistently more errors on the injured side. SEBT scores were close between injured and healthy ankles (<1 percentage point difference), with modest improvement across sessions. SHT and F8T completion times ranged from 10 to 14 s and showed only minimal change across sessions, with overlapping distributions between injured and healthy ankles. Given the absence of inferential testing, we describe these patterns as small or minimal numerical differences rather than as the absence of statistically significant differences.

**Figure 6:**
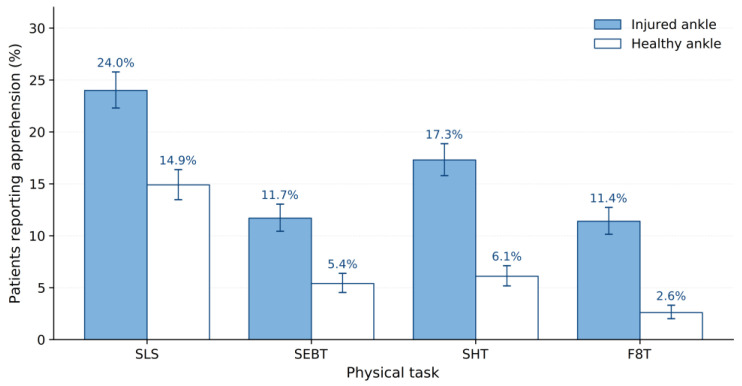
**Apprehension during physical tasks.** *Proportion of patients reporting apprehension per task (each task distinguished by colour and pattern), injured versus healthy ankle. The Single-Leg Stance elicited the highest apprehension, followed by the Side Hop Test; apprehension was systematically higher on the injured side.*

Figure 6 shows the proportion of patients reporting apprehension during each physical task, with each task delineated by colour and pattern. The SLS elicited the highest apprehension rates, followed by the SHT. Apprehension was systematically higher on the injured ankle than on the healthy ankle, regardless of whether one or both ankles were assessed.

### 3.5. Return to Sport

**Figure 7:**
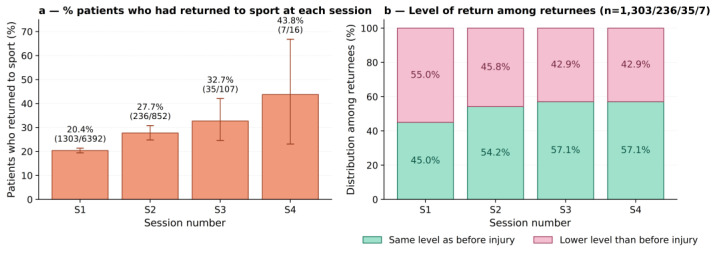
**Return to sport relative to the first assessment.** *Overall, 20.4% of patients (95% CI, 19.4–21.4) had already returned to sport before their first Ankle-GO assessment. The proportion reporting a return to their previous level increased across sessions, from 45.0% to 57.1%. Later-session estimates are based on small sample sizes and should therefore be interpreted cautiously.*

A central and clinically important finding of this analysis is that 20.4% (95% CI 19.4–21.4) of patients had already returned to sport before their first Ankle-GO assessment (Figure 7). Among them, 45.0% (95% CI 42.3–47.7) reported having regained their previous level of performance at session 1; this proportion rose to 54.2% at session 2, 57.1% at session 3, and 57.1% at session 4. The overall proportion of patients reporting any return to sport increased from 20.4% at session 1 to 43.8% at session 4, though the latter estimate is based on only 16 patients (95% CI 23.1–66.8).

## 4. Discussion

This study provides the first large-scale description of how the Ankle-GO composite score is used in routine clinical practice. Three descriptive observations emerge: (i) 20.4% of patients had already returned to sport before their first Ankle-GO assessment, directly addressing the pre-specified aim of quantifying prospective versus retrospective use; (ii) repeat assessments are rare (14% of all sessions), severely limiting any inference about individual recovery trajectories from the current dataset; and (iii) substantial heterogeneity in assessment timing, injury type, and operator practices generates score distributions that should not be used to derive new clinical cut-offs but rather to inform the design of future prospective studies.

### 4.1. The Intended-Versus-Actual-Use Gap

The finding that 20.4% of patients had returned to sport before their first Ankle-GO session is directly addresses one of the pre-specified aims of this study. The Ankle-GO was developed to inform RTS decisions prospectively [12], yet a substantial proportion of practitioners appear to use it as a retrospective documentation tool. Several unmeasured factors may hypothetically contribute to this pattern, including the absence of widespread structured rehabilitation pathways for ankle injuries [11,23,24], the time pressure of clinical practice, and patient-driven return to activity before completion of formal rehabilitation. Another possible explanation is that many patients return to sport before the two-month time point commonly used in Ankle-GO studies. In this context, the >8 cut-off may be applied after the RTS decision has already been made, thereby limiting its practical value as a prospective decision-making tool. This does not necessarily invalidate the cut-off, but highlights that its clinical usefulness is highly dependent on assessment timing. This gap is consistent with recent reports that physiotherapists’ adherence to ankle-sprain clinical practice guidelines remains heterogeneous and incomplete [25]. Closing this gap will require not only further validation of the Ankle-GO itself, but also implementation-science approaches addressing clinician training, patient education, and integration of structured assessment into existing rehabilitation pathways.

### 4.2. Implementation Variability and Data Quality

Approximately 23% of all registered patients were excluded from the analytic cohort, primarily because of implausible measurements, which accounted for 68.5% of all exclusions. This rate of exclusion is high and indicates that data quality requires further attention in routine Ankle-GO implementation. However, the database does not allow us to determine whether implausible values resulted from assessor-related factors, data-entry errors, form design issues, ambiguous instructions, or system-related problems. Accordingly, the impact of assessor training and quality-control procedures on data quality should be evaluated prospectively before drawing firm conclusions about optimal implementation conditions. The very high proportion of missing demographic data (56% for sex) likely reflects the fact that several fields were not made mandatory in the early versions of the web application; this has since been corrected, but the data captured during the study period limit subgroup analyses. We therefore caution against direct use of the present descriptive statistics to derive new cut-offs or expected values, and underline the need for prospective registries with mandatory data fields and standardised assessor training.

The coexistence of bilateral and unilateral testing without explicit selection criteria introduces additional methodological heterogeneity. Mean scores on the injured ankle were consistently lower in unilateral sessions than in bilateral sessions; however, this descriptive difference should be interpreted cautiously because the database does not allow us to determine whether it reflects patient selection, clinical severity, assessor practices, or other unmeasured factors. Similarly, mean healthy-ankle scores in this dataset were lower than the value of 19.6 reported in the original validation cohort [12], a phenomenon possibly explained by pain inhibition, fear of falling on the injured side, and partial deconditioning of the unaffected limb [28,29]. The increase in healthy-ankle scores between sessions 1 and 2 may partly reflect learning or familiarisation effects rather than true physiological change. This is particularly plausible in a real-world setting without centralized assessor training or a standardized familiarisation procedure, and reinforces that between-session changes should be interpreted descriptively.

### 4.3. Score Distributions and Subtest Behaviour

Mean Ankle-GO scores on the injured ankle at the first session (≈15–16 points) were higher than those previously reported in prospective LAS cohorts at four months (14.2 points) [12], and substantially higher than those reported in surgical cohorts (10.3 after ligament reconstruction at four months; 10.7 after Achilles repair at six months) [19,20]. These differences may be partly explained by heterogeneous timing of the first assessment and by the predominance of non-surgical sprain patients in our cohort, but they remain descriptive and should not be interpreted as evidence of pathology or timing specific effects. They reinforce the message that pathology- and time-specific cut-offs derived from controlled cohorts cannot be applied to this heterogeneous, non-standardised dataset [12,19,20,21].

Across subtests, most first-session results were below the published RTS cut-off values for the SEBT, SHT, and F8T, suggesting that many patients had not yet met functional criteria for safe return [11]. Conversely, mean FAAM and ALR-RSI scores were closer to or above the corresponding cut-offs. These contradictory patterns across subtests reinforce the original rationale for a composite score: no single test captures all dimensions of recovery. Of note, ALR-RSI improvements across sessions were smaller than those observed for the FAAM and physical tasks, suggesting that psychological readiness recovers more slowly than physical function and may partly explain why some patients return to sport despite suboptimal psychological scores [30].

The moderate correlation between sessions 1 and 2 (ρ = 0.46) indicates that the score is informative but not deterministic at the individual level, reinforcing the value of repeated rather than single-point assessments. Establishing a robust minimum detectable change from a dedicated test–retest reliability study is now an explicit priority, as a correlation coefficient alone does not allow clinicians to determine whether an individual patient has truly improved, remained stable, or worsened between two assessments.

### 4.4. Comparison with External Data

Return-to-sport rates observed here (20% at session 1, rising to 44% at session 4) are lower than those reported in surgical cohorts (76.5% at six months after ligament reconstruction in one independent series [31]; 80% in recreational and 48% in competitive athletes after arthroscopic stabilisation [32]). Several factors likely account for these differences, including the predominance of conservatively treated sprains in our cohort, variable timing of assessments, and the possibility that fully recovered patients did not return for additional physiotherapy sessions. We acknowledge that a substantial proportion of our reference list draws on work from our own group, which reflects the recent emergence of the Ankle-GO; we have therefore deliberately incorporated external comparators where available [22,25,31,32], and we explicitly identify independent replication as a future priority.

### 4.5. Limitations

This study has several important limitations. First, this is a purely descriptive analysis of non-standardised real-world data; no inferential testing was performed and no claim of validation should be inferred. Second, the cohort is heterogeneous (sprains, fractures, surgery, Achilles repair), and timing between injury and first assessment was highly variable, precluding pathology-specific or time-specific conclusions; pooling was a deliberate choice to characterise actual practice but limits clinical interpretability. Although the delay between injury and first assessment was reported using the median and IQR, analyses stratified by time since injury were not performed and should be considered in future prospective studies. For the same reason, observed differences between groups or session types should be regarded as exploratory and descriptive only, because no adjusted analyses were performed to account for differences in pathology, time since injury, sport level, or assessment context. Third, only 14% of sessions corresponded to follow-up assessments, so the apparent improvement across sessions reflects largely between-cohort rather than within-patient change. In addition, the database did not capture the reasons for retrospective Ankle-GO use after sport resumption; therefore, explanations such as clinician time pressure, lack of structured rehabilitation pathways, or patient-driven return to activity remain speculative. Fourth, missing demographic data were substantial (56% for sex), and selection bias linked to exclusion of patients with implausible values cannot be ruled out. Fifth, no centralised assessor training or formal inter-rater reliability assessment was conducted assessments were performed by an unknown number of physiotherapists across multiple countries and several subtests are operator-dependent. Because assessor training level was not recorded, we could not assess its relationship with data quality or determine the training requirements needed for optimal routine implementation of the Ankle-GO. Sixth, the geographical concentration of the cohort in France (92.5%) limits generalisability to other healthcare systems and rehabilitation pathways. Seventh, several authors are developers of the Ankle-GO and the study was supported by Ramsay Santé; we have aimed to discuss these limitations openly, but the reader should bear this conflict of interest in mind. Finally, the minimum clinically important difference (MCID) for the composite Ankle-GO score has not yet been formally established, limiting both cross-sectional and longitudinal interpretation.

These findings should be interpreted in light of the geographical distribution of the cohort, which was mainly based in France, Switzerland, and Belgium, and may therefore not be directly generalizable to other countries or healthcare systems.

Future work should address these limitations through prospective cohorts with standardised assessment timing, mandatory data fields, structured assessor training, blinded outcome assessment, and pathology-specific stratification, together with formal test–retest reliability studies to derive a robust MCID. Independent external replication of the Ankle-GO’s psychometric properties by groups unconnected to its development is an explicit priority.

## 5. Conclusions

In this analysis of 6934 patients from a large French-speaking real-world database, the Ankle-GO composite score was applied to a heterogeneous population of ankle injuries, but its real-world implementation diverged substantially from its intended prospective use: one in five patients had already returned to sport before their first assessment, and only 14% of sessions corresponded to follow-up evaluations. Importantly, the present data should not be interpreted as evidence of country-specific effectiveness of the Ankle-GO within return-to-sport protocols. Although the web application was used in several countries, the cohort was predominantly French-speaking and geographically concentrated in France, with smaller contributions from Belgium and Switzerland. Moreover, no standardised return-to-sport protocol, prospective reinjury surveillance, or country-level implementation framework was embedded in the database. Therefore, this study describes real-world uptake and implementation patterns rather than comparative effectiveness across healthcare systems. Future international prospective studies should evaluate whether structured Ankle-GO-based return-to-sport pathways improve clinical decision-making, reinjury rates, and return-to-sport outcomes compared with usual care. Repeat assessments were rare, missing demographic data were substantial, and timing between injury and assessment was highly variable. These observations carry three concrete messages for the field: (i) the Ankle-GO is now widely adopted in French-speaking countries and is feasible in routine clinical practice; (ii) current real-world data are not suitable for deriving new cut-offs or for inferring recovery trajectories; and (iii) future prospective studies must standardise assessment timing, enforce mandatory data fields, train assessors centrally, and stratify by pathology before the Ankle-GO can be regarded as a validated RTS decision-support tool. Closing the gap between intended and actual use of the score is at least as important as further psychometric refinement, and will require coordinated efforts across clinicians, researchers, and tool developers.

## Figures and Tables

**Table 1:** Baseline characteristics of the analytic cohort (*n* = 6934).

Characteristic	Value	Missing, %
Age, years, mean ± SD (range)	26.0 ± 10.0 (10–68)	—
Age < 18 years, %	23.1	—
Body-mass index, kg/m^2^, mean ± SD	22.8 ± 3.2	—
Sex (*n* = 3038 with data)		56.2
Male, % (95% CI)	62.6 (60.9–64.3)	
Female, % (95% CI)	37.4 (35.7–39.1)	
Country of treatment		—
France, %	92.5	
Belgium, %	4.9	
Switzerland, %	2.2	
Assessment indication		—
Lateral ankle sprain, % (95% CI)	88.5 (87.6–89.3)	
Fracture, %	4.1	
Ankle stabilisation surgery, %	3.7	
Achilles tendon repair, %	1.0	
Other, %	2.7	
Previous ankle sprain, % (approx.)	≈23	—
Sport participation, %	82.4	—
Professional, %	6.1	
Competitive, %	39.3	
Recreational, %	37.1	
Delay injury → first assessment, months, median (IQR)	2.33 (1.31–4.07)	—

SD, standard deviation; CI, confidence interval (Wilson); IQR, interquartile range. Sex proportions are reported only over the *n* = 3038 patients with sex recorded and are not extrapolated to the full cohort. “Other” indication is the residual after the listed categories. Dashes (—) indicate fields with no missing-data flag reported.

## Data Availability

The data underlying this study contain potentially identifying patient information and cannot be made publicly available. Anonymised aggregated data are available from the corresponding author on reasonable request and subject to compliance with applicable data-protection regulations.

## Abbreviations

The following abbreviations are used in this manuscript:

ADL	Activities of daily living
ALR-RSI	Ankle Ligament Reconstruction–Return to Sport after Injury (scale)
AUC	Area under the curve
CAI	Chronic ankle instability
CI	Confidence interval
F8T	Figure-of-8 hop Test
FAAM	Foot and Ankle Ability Measure
GDPR	General Data Protection Regulation
ICC	Intraclass correlation coefficient
IQR	Interquartile range
LAS	Lateral ankle sprain
MCID	Minimum clinically important difference
MDC	Minimum detectable change
RTS	Return to sport
SD	Standard deviation
SEBT	Star Excursion Balance Test
SHT	Side Hop Test
SLS	Single-Leg Stance Test
STROBE	Strengthening the Reporting of Observational Studies in Epidemiology
